# Cholesterol Induces Pyroptosis and Matrix Degradation *via* mSREBP1-Driven Endoplasmic Reticulum Stress in Intervertebral Disc Degeneration

**DOI:** 10.3389/fcell.2021.803132

**Published:** 2022-01-31

**Authors:** Jiansen Yan, Shuangxing Li, Yangyang Zhang, Zhihuai Deng, Jiajun Wu, Zhengqi Huang, Tianyu Qin, Yin Xiao, Jie Zhou, Kang Xu, Wei Ye

**Affiliations:** ^1^ Guangdong Provincial Key Laboratory of Malignant Tumor Epigenetics and Gene Regulation, Sun Yat-Sen Memorial Hospital, Sun Yat-Sen University, Guangzhou, China; ^2^ Department of Spine Surgery, Sun Yat-Sen Memorial Hospital of Sun Yat-Sen University, Guangzhou, China; ^3^ Institute of Health and Biomedical Innovation, Faculty of Science and Engineering, Queensland University of Technology, Brisbane, QLD, Australia; ^4^ Australia-China Centre for Tissue Engineering and Regenerative Medicine, Queensland University of Technology, Brisbane, QLD, Australia; ^5^ Department of Breast Surgery, Affiliated Cancer Hospital and Institute of Guangzhou Medical University, Guangzhou, China

**Keywords:** intervertebral disc degeneration, cholesterol, endoplasmic reticulum stress, SREBP1, nucleus pulposus cell, pyroptosis

## Abstract

Intervertebral disc degeneration (IDD) is closely associated with low back pain, but its underlying mechanism remains unclear. Cholesterol is an essential nutrient in mammalian cells. Alterations in cholesterol levels lead to impairments in cell physiology, such as cell proliferation and signal transduction. Previous clinical studies demonstrated that hypercholesterolemia could be a potential risk factor for IDD, but how cholesterol induces IDD remains unknown. The current study aimed to explore the regulatory role of cholesterol in IDD development and the potential underlying mechanisms. It was found that different forms of cholesterol levels were elevated in degenerative nucleus pulposus (NP) tissues in both humans and Sprague–Dawley rats. Rats fed a high cholesterol diet (HCD) exhibited degenerative features in the lumbar intervertebral disc compared with those fed a standard diet. Interestingly, this effect could be abolished by cholesterol-lowering drug atorvastatin. In NP cells treated with TNF-α and IL-1β, a significantly higher level of cholesterol was observed. These results suggested a pivotal role of cholesterol in the progression of IDD. We also observed accelerated pyroptosis in NP cells and extracellular matrix (ECM) degradation in the rat NP cells treated with exogenous cholesterol. We further demonstrated that endoplasmic reticulum stress was responsible for cholesterol-induced pyroptosis and ECM degradation. Moreover, RNA-seq analysis revealed that the mature form of SREBP1 (mSREBP1), an important regulator of lipid metabolism, is involved in regulating endoplasmic reticulum stress in knockdown experiments. In conclusion, this study demonstrated that cholesterol could induce pyroptosis in NP cells and ECM degradation by activating endoplasmic reticulum stress through stimulating mSREBP1 in IDD.

## Introduction

Low back pain (LBP) is a common musculoskeletal disorder and the most predominant cause of disability (Knezevic et al., 2021). The prevalence of LBP has been continuously rising, particularly in working-age groups, from 1990 to 2015 globally (Hartvigsen et al., 2018), and LBP induces tremendous social and economic burdens. Although the etiology and pathology are complicated and not well understood, IDD is believed to play a vital role in the process of LBP (Livshits et al., 2011). In the human body, the intervertebral disc is the largest avascular organ and consists of a gel-like nucleus pulposus (NP) in the center, a concentric anulus fibrosus (AF) and superior and inferior cartilaginous endplates (Urban and Roberts, 2003). During IDD, NP tissues undergo the most dramatic changes and are characterized by dysfunctional and reduced numbers of NP cells, as well as degradation of the extracellular matrix (ECM), including collagen II (COL2A) and aggrecan. NP cells, which are the predominant cell type within the NP region, regulate ECM homeostasis by controlling anabolism and catabolism. With disc degeneration, excessive degradation and decreased synthesis of ECM compromise the capacity of NP tissues to retain water, thus affecting the biomechanical properties of discs in response to load (Adams and Roughley, 2006). Therefore, delineating the mechanisms by which the homeostasis of ECM metabolism and NP cell survival are regulated is crucial in comprehensively understanding the pathological mechanisms of IDD.

Similar to IDD, hypercholesterolemia is also a global public health problem because of high-fat diets and inactive lifestyles, and this condition is closely linked to diseases such as cancer (Buchwald, 1992), atherosclerosis (Ference et al., 2017), and type 2 diabetes (Perego et al., 2019), posing an enormous threat to human health (NCD-RisC, 2020). Apart from being an important component of mammalian cell membrane structure, cholesterol also serves as the precursor for the biosynthesis of bile acids and steroid hormones. Metabolic homeostasis of cellular cholesterol is maintained by fine tuning the intricate interplay between *de novo* synthesis, uptake, efflux and storage (Luo et al., 2020). Recently, several epidemiological studies have revealed a potential association between metabolic disturbances in cholesterol and the risk of IDD or LBP in both Western (Longo et al., 2011) and Eastern countries (Zhang et al., 2016; Yoshimoto et al., 2018). Besides, statins, a widely used drug class for lowering blood cholesterol, have been shown to alleviate IDD *in vivo* and *in vitro* (Hu et al., 2014; Tu et al., 2017). Moreover, APOE-knockout rabbits, which are common animal models for studying atherosclerosis with disrupted lipid metabolism, exhibited more severe disc degeneration and fewer cells in NP tissues than wild-type rabbits (Beierfuß et al., 2019). While studies have shown a possible relationship between cholesterol and disc degeneration, whether cholesterol induces IDD and its underlying molecular mechanisms still remain unknown.

Programmed cell death has long been confirmed to contribute to the progression of IDD (Ding et al., 2013; Zhou et al., 2019). Distinct from apoptosis, pyroptosis is an inflammatory type of programmed cell death characterized by gasdermin-mediated rupture of the plasma membrane and the leakage of proinflammatory cytokines (Liu et al., 2016). Our previous study found that TNF-α activated NLRP3 inflammasome through the NF-κB signaling pathway in NP cells, and this effect could be alleviated by the cholesterol-lowering drug atorvastatin (Chen et al., 2021). In addition, pyroptosis-associated biomarkers including NLRP3, cleaved caspase-1 (p20) and IL-1β are prominently expressed in NP tissues in IDD (Zhao et al., 2020). Cholesterol and its metabolites have been reported to promote activation of NLRP3 inflammasome in arthrosclerosis (Duewell et al., 2010; Guo et al., 2018). *In vivo*, a high fat and high cholesterol diet could induce pyroptosis in mice with nonalcoholic steatohepatitis (Koh et al., 2020), suggesting a close relationship between cholesterol and pyroptosis. Therefore, deciphering whether cholesterol is involved in the induction of pyroptotic cell death during IDD is of great importance.

The endoplasmic reticulum (ER) is responsible for lipid biosynthesis, calcium storage and protein folding (Binet and Sapieha, 2015). Many studies have revealed that persistent ER stress could induce programmed cell death, especially apoptosis (Liao et al., 2019; Luo et al., 2019). In addition, a recent research also reported ER stress was strongly associated with pyroptosis in human trophoblasts (Cheng et al., 2019). However, it is unclear whether cholesterol induces IDD by activating ER stress in NP cells.

The objective of the present study was to explore the role of cholesterol in IDD. We hypothesized that cholesterol could induce pyroptotic cell death and promote matrix degradation in IDD. Moreover, an HCD could induce IDD in rats, and atorvastatin could reverse this effect. In addition, cholesterol could induce IDD by activating ER stress in NP cells.

## Materials and Methods

### Collection of Patient Samples

40 NP tissues were collected from 34 patients (19 females and 15 males, aged from 23 to 75 years) who underwent spine surgeries at Sun Yat-Sun Memorial Hospital from 2019 to 2021. All patients in this study underwent posterior discectomy where posterior and lateral AF was incised at least 1 cm depth by scalpel and the inner NP tissues were then collected with laminectomy rongeur. Samples were fixed in 4% paraformaldehyde for histological staining or stored at −80°C immediately for cholesterol analysis. The degree of disc degeneration was assessed by Pfirrmann classification based on T2-weighted MRI scans before surgery. This study protocol was approved by the Ethics Committee of Sun Yat-Sen Memorial Hospital [No. (2020) 233]. All patients signed informed consent.

### Animals and Diets

All animal experiments were performed according to the guidance of the Institutional Animal Care and Use Committee (IACUC) of Sun Yat-Sen University (No. SYSU-IACUC-2020-B0165). Sprague-Dawley (SD) rats were obtained from the Laboratory Animal Center of Sun Yat-Sen University and were kept in a specific pathogen-free environment with a 12 h light and 12 h dark regime and sufficient experimental food and water. Male SD rats (n = 36, 10 weeks old, 320–350 g) were randomly separated into three groups. The rats in control group (n = 12) were put on a standard chow diet, and the rest rats were fed a high cholesterol diet (HCD, BiotechHD, Beijing, China) containing 92.5% standard diet food, 2.5% cholesterol and 5% lard. After 4 weeks of HCD feeding, 12 rats were randomly selected and treated with atorvastatin (20 mg/kg/day, oral gavage) (Lipitor, Pfizer, United States) in addition to HCD feeding for an additional 8 weeks. Body weight was recorded weekly, and serum lipid levels were measured by an automatic biochemistry instrument (Beckman Coulter, United States) every 4 weeks. The rats were euthanized, and the lumbar spine was dissected to obtain vertebral body-disc-vertebral body specimens for histological staining. Degeneration degree of intervertebral discs were evaluated based on grading system established by Masuda et al. (2005).

### Surgery-Induced IDD Model

Fourteen male SD rats (10 weeks old, 320–350 g) were obtained for the establishment of IDD model. Among them, six rats underwent AF needle puncture, and eight rats were left intact. General anesthesia was administered by intraperitoneal injection of 2% pentobarbital. The location of two intervertebral discs (Co7/8 and Co8/9) was identified by palpating processes on the coccygeal vertebrae. After sterilizing tail skin with 75% ethanol twice, the middle point of intervertebral disc of Co7/Co8 and Co8/Co9 was penetrated vertically with a 20-gauge needle from dorsal to ventral. The needle was inserted 5 mm into the disc and then rotated 360° clockwise steadily and kept for 30 s before being extracted. Tail discs were harvested 4 weeks after surgery; those (discs at Co7/Co8) used for histological staining were fixed in 4% paraformaldehyde, those (discs at Co8/Co9) used for cholesterol analysis were frozen at −80°C immediately. To collect NP tissues of punctured discs, A circle that is comparable to normal NP tissues was drawn and incised with a sharp No.11 blade and tissues in this circle were collected with a Dumont tweezer.

### Rat NP Cell Isolation and Culture

Rat NP cells were isolated and cultured as previously reported (Huang et al., 2020). After SD rats (male, weight 200–250 g) were euthanized, NP tissues were harvested from caudal intervertebral discs, and then digested in 0.2% pronase (Sigma-Aldrich, United States) for 45 min at 37°C, followed by 2.5% collagenase II (C8150, Solarbio, Beijing, China) digestion for 15 min at 37°C. Subsequently, the digested tissues were washed and cultured in DMEM (Gibco, United States) containing 10% FBS (Gibco, United States) and 1% antibiotics (P1400, Solarbio, Beijing, China) at 37°C. Cells were passaged until they reached 80–90% confluence. Water-soluble cholesterol (Sigma-Aldrich, C4951) was used to treat NP cells.

### Immunohistochemistry

For rat NP tissues, the specimens were fixed with 4% paraformaldehyde for 48 h after careful dissection, decalcified in EDTA on a slow shaker for 8 weeks at room temperature, embedded in paraffin and cut into sections of 4-µm thickness. For human NP tissues, decalcification was not necessary. Subsequently, the slides were deparaffinized with xylene and rehydrated in graded ethanol series, followed by antigen retrieval with pepsin for 20 min at 37°C for rat tissues or with EDTA retrieval buffer (ZLI-9068, Zhongshanjingqiao Biotechnical, Beijing, China) for 10 min at 100°C in microwave oven for human tissues. Endogenous peroxidase activity was quenched by 3% hydrogen peroxide. Then, the slides were blocked with goat serum for 30 min and incubated with PBS as a negative control or the following primary antibodies overnight at 4°C: anti-NLRP3 (1:200, A12694, ABclonal), anti-p20 (1:200, AF4005, Affinity), anti-CHOP (1:200, ab11419, Abcam), anti-GRP78 (1:200, ab21685, Abcam), anti-COL2A (1:100, ab34712, Abcam), anti-Aggrecan (1:100, ab36861, Abcam), anti-MMP13 (1:200, ab39012, Abcam), and anti-SREBP1 (1:200, ab28481, Abcam). The sections were incubated with biotin-labeled secondary antibody for 30 min at room temperature and HRP-conjugated streptavidin for 20 min and then developed with DAB solution (Zhongshanjingqiao Biotechnical). The sections were counterstained with 1% hematoxylin, mounted, photographed under a microscope.

### Real-Time Polymerase Chain Reaction and RNA Sequencing

Total RNA was extracted in rat NP cells with RNAiso Plus (Takara, Japan) in accordance with the manufacturer’s protocols. Reverse transcription was performed using 500 ng of total RNA and a Prime Script RT Master Mix kit (Takara). Real-time quantitative PCR was performed with a LightCycler 96 System (Roche) using SYBR Green Master Mix (Yeasen, Shanghai, China) in accordance with the manufacturer’s protocols. Data from triplicates were collected and analyzed using the 2^−ΔΔCt^ method. β-actin expression was used to normalize the expression of all genes. The primers used in this study were listed in Supplementary Table S1.

For RNA sequencing, total RNA was extracted from rat NP cells stimulated with PBS or 10 μg/ml cholesterol. cDNA was then generated and analysed by Illumina HiSeq 2000 platform. The datasets presented in this study can be found in online repositories: https://www.ncbi.nlm.nih.gov/sra/PRJNA783219.

### Knockdown of SREBP1 by shRNA

Both shRNA (SREBP1) and corresponding negative control were obtained from HanBio Technology Company (Shanghai, China). The culture medium was replaced with regular medium after 3 days of transfection. The transfection efficiency was assessed by western blot and fluorescence after 5–7 days of transfection. The sequence of shRNA-SREBP1-a was 5′- CAG​CTC​TCC​TGA​GAG​CTT​CTC​TTC​T-3′, the sequence of shRNA-SREBP1-b was 5′- CCC​GAC​TAT​TCT​GTG​AAC​ATC​TCC​T-3′, the sequence of shRNA-SREBP1-c was 5′- CGG​GAC​AGC​TTA​GCC​TCT​ACA​TCA​A-3′, and the sequence of shRNA-control was 5′- TTC​TCC​GAA​CGT​GTC​ACG​TAA -3′.

### ELISA

Culture supernatants of rat NP cells were collected following treatments. Concentrations of IL-1β were tested by ELISA in accordance with the manufacturer’s protocols (Dakewei, Shenzhen, China). Three experimental replicates were analyzed for each condition.

### TEM

Rat NP cells were trypsinized, collected into pellets, and fixed with 2.5% glutaraldehyde overnight at 4°C. After being washed with PBS, the pellets were postfixed in 1% osmium tetroxide for 2 h at 4°C and then washed again in buffer, after which they were dehydrated through an ascending graded ethanol series. Subsequently, samples were fully infiltrated and embedded in Epon 812. Ultrathin sections (100 nm) were prepared with a Leica UC7 ultramicrotome, poststained with uranyl acetate and lead citrate, and visualized with a FEI Tecnai G2 Spirit transmission electron microscope (FEI company, United States).

### Filipin Staining

Filipin is a polyene macrolide antibiotic with ability of binding to free cholesterol via hydrophobic interactions and is inherently fluorescent. For rat NP cells, samples were washed with PBS prior to fixation in 4% paraformaldehyde and then incubated with a 50 μg/ml filipin working solution (Filipin complex, MCE, United States) for 2 h in the dark. For human NP tissues, frozen sections were washed in PBS and fixed in 10% formaldehyde for 1 h. The fixed sections were rinsed twice with PBS and then incubated with a 0.25 mg/ml filipin solution at 4°C overnight in the dark. Cell samples were viewed by line scanning confocal microscopy, and tissue samples were imaged by fluorescence microscopy (IX73, Olympus, Japan).

### Detection of Cholesterol Level

Cellular and tissue cholesterol levels were tested with a Total Cholesterol Assay Kit (Cell Biolabs, United States). NP cells and tissues were homogenized by a freezing bead homogenizer. The cholesterol was extracted by 200 μl extracting solution containing 77.7 μl chloroform, 121.2 μl isopropanol and 1.1 μl NP-40 for 20 min. The extracts were air dried at 60°C in oven for 30 min and then put under vacuum for 2 h to remove remaining solvents. The amount of total cholesterol and free cholesterol was detected in accordance with the manufacturer’s protocols. The level of cholesterol ester was obtained by subtracting free cholesterol from total cholesterol.

### LDH Assay

LDH release of NP cells were determined with the CytoTox96 nonradioactive cytotoxicity assay (Promega, United States). Cells were plated and cultured in phenol red-free medium. Following treatments, the culturing medium was collected, and LDH release was assessed in accordance with the manufacturer’s recommendation.

### Sulfated Glycosaminoglycan Assay

Cells were plated, treated and incubated in phenol red-free medium. Following treatments, the medium was collected, and sGAG was assessed by a Sulfated Glycosaminoglycan Assay Kit (Biocolor, United Kingdom) in accordance with the manufacturer’s recommendation.

### Immunofluorescence

Cells were cultured in 24-well plate. Following treatments, samples were fixed in 4% paraformaldehyde,permeabilized in 0.5% Triton X-100 (T8200, Solarbio, Beijing, China)and blocked with 5% goat serum, followed by incubation with anti-COL2A (1:100, YT1022, Immunoway), anti-GSDMD (1:100, A18281, ABclonal) and anti-GRP78 (1:200, ab21685, Abcam) overnight at 4°C. Subsequently, samples were incubated with secondary antibodies (Invitrogen, United States) for 1 h in the dark. Cell nuclei staining was performed with DAPI (Solarbio, China) and photographed immediately with a fluorescence microscope (IX73, Olympus, Japan).

### Protein Extraction and Western Blotting

After the treatments, the cells were lysed on ice for 20 min with a mixture of 100 μl RIPA buffer (Fdbio science, Hangzhou, China), 1 μl protease inhibitor (Fdbio science) and 1 μl phosphatase inhibitor (Fdbio science) and centrifuged at 14,000 g for 25 min. Protein concentrations were determined using a BCA Total Protein Assay Kit (CWBIO, China). Protein samples (40 μg/lane) were separated on SDS-PAGE gels (8% or 12%, Epizyme, China) and electrotransferred to PVDF membranes. The blots were incubated with the following primary antibodies: anti-NLRP3 (1:500, A12694, ABclonal), anti-GSDMD (1:500, 39754, CST), anti-p20 (1:500, AF4005, Affinity), anti-CHOP (1:500, ab11419, Abcam), anti-GRP78 (1:500, ab21685, Abcam), anti-COL2A (1:500, ab34712, Abcam), anti-Aggrecan (1:500, ab36861, Abcam), anti-MMP3 (1:500, ab52915, Abcam), anti-MMP13 (1:500, ab39012, Abcam), anti-ADAMTS5 (1:1000, ab41037, Abcam), and anti-SREBP1 (1:1000, ab28481, Abcam). The membranes were washed and incubated with the HRP-conjugated secondary antibodies (1:8000, ABclonal, China) for 1 h. The bands were developed with chemiluminescence reagents and imaged by a myECL imager (Syngene G:BOX ChemiXT4, United Kingdom). The experiment was repeated in triplicate, and the blots were quantified by ImageJ software. An antibody against β-actin (AC026, 1:1000; ABclonal) served as an endogenous control.

### Statistical Analysis

All data were analyzed from at least three independent experiments, presented as the mean ± standard deviation (SD) and analyzed using GraphPad Prism 8 (GraphPad Software, CA). The values between two groups were assessed by unpaired two-tailed Student’s t test, and differences among the multiple groups were analyzed by one-way ANOVA, and *p* value < 0.05 was considered statistically significant.

## Results

### Cholesterol Levels Were Elevated in Degenerative NP Tissues and Inflammatory Cytokine-Treated Rat NP Cells

**FIGURE 1:**
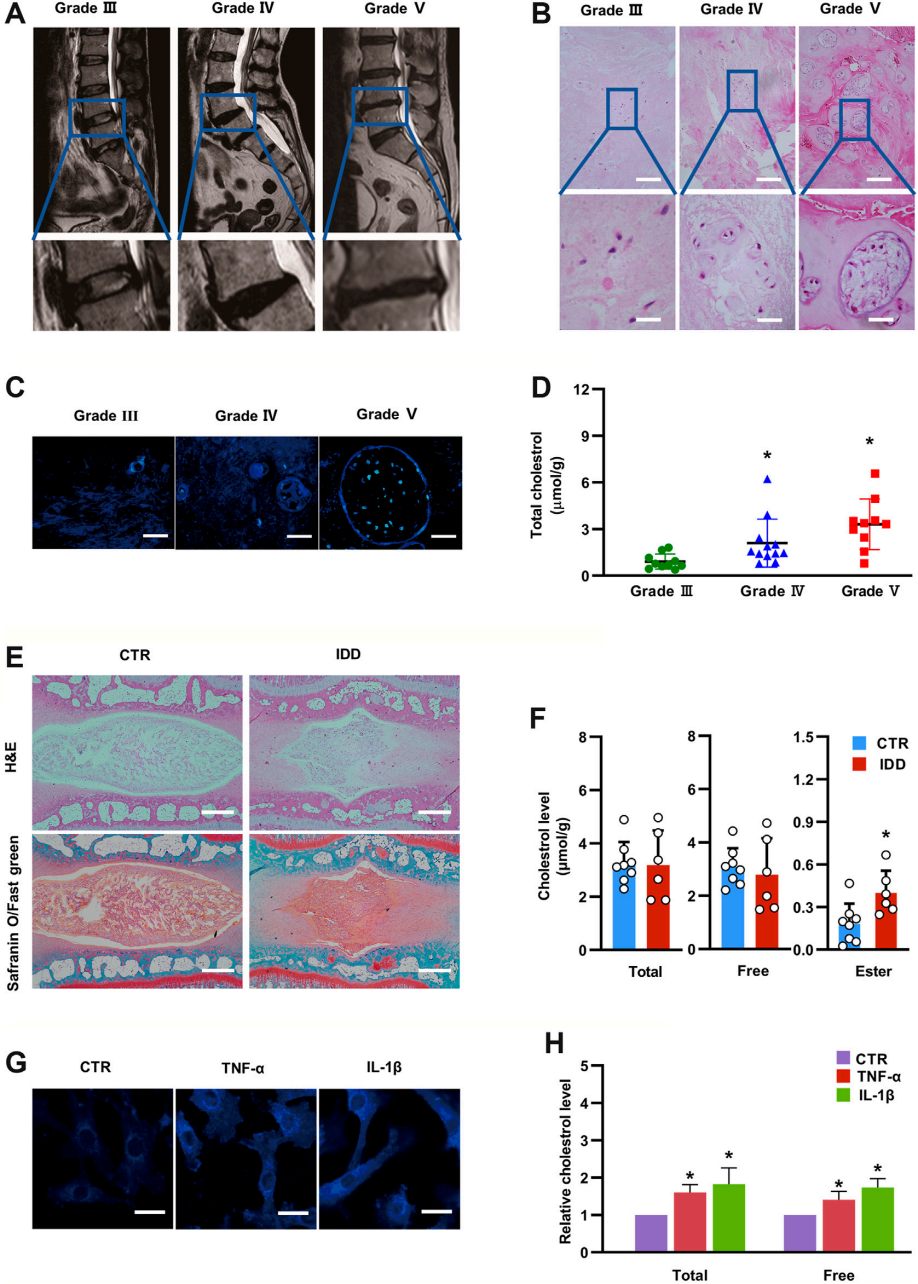
Cholesterol levels are elevated in degenerative NP tissues and inflammatory cytokine-treated rat NP cells. **(A)** Lumbar MRI scans showing different degrees of IDD. **(B)** H&E staining of degenerative NP tissues from patients (upper panel, bar = 200 μm; lower panel, bar = 50 μm). **(C)** Filipin staining to detect free cholesterol in degenerative NP tissues from patients (bar = 50 μm). **(D)** Total cholesterol levels were elevated in degenerative human NP tissues with increased severity (n = 10–12). **(E)** Representative H&E and Safranin O/Fast green staining of rat intervertebral discs (bar = 500 μm). **(F)** Levels of total cholesterol, free cholesterol and cholesterol ester in punctured rat NP tissues and control NP tissues (n = 6–8). **(G)** Filipin staining of NP cells showing increased free cholesterol levels after TNF-α (50 ng/ml) and IL-1β (10 ng/ml) stimulation (bar = 30 μm). **(H)** The levels of total cholesterol and free cholesterol were elevated in NP cells treated with TNF-α (50 ng/ml) or IL-1β (10 ng/ml) for 24 h (n = 3). The data are presented as the mean ± SD. *p < 0.05.

To explore the relationship between IDD and cholesterol, we first collected 32 lumbar NP tissues with different degrees of degeneration (Pfirrmann grading system) according to lumbar MRI scans (Figure 1A). The NP samples included 10 Grade III samples, 12 Grade IV samples and 10 Grade V samples (Details were presented in Supplementary Table S2). Hematoxylin and eosin (H&E) staining indicated decreased cell numbers but more aberrant cell clusters in NP tissues with higher Pfirrmann grades (Figure 1B), which was consistent with the MRI scan results. Filipin, a polyene macrolide antibiotic, has been widely used to localize and quantitate free cholesterol due to its ability to fluoresce and bind to cholesterol (Muller et al., 1984). Histological filipin staining demonstrated an increase in free cholesterol in degenerative NP tissues with higher Pfirrmann grades (Figure 1C). In addition, total cholesterol levels were also significantly elevated in degenerative NP tissues with increasing severity (0.91 ± 0.49 μmol/g in Grade III, 2.11 ± 1.54 μmol/g in Grade IV, 3.31 ± 1.62 μmol/g in Grade V) (Figure 1D). Moreover, an IDD model was established in SD rats by needle puncture of two-level caudal discs (Co7/8 and Co8/9). The discs (Co7/8) were harvested at 4 weeks after the needle puncture and were subjected to H&E and safranin-O/fast green staining to confirm the success of the IDD model (Figure 1E). The levels of cholesterol esters in degenerative NP tissues (Co8/9) were significantly increased by approximately twofold relative to that in the control group (0.40 ± 0.16 μmol/g in the IDD group vs. 0.18 ± 0.14 μmol/g in the control group), although total cholesterol and free cholesterol levels were not significantly different between the two groups (Figure 1F). In addition, both TNF-α and IL-1β, which are the major increased proinflammatory cytokines in degenerative disc tissues, were used to stimulate NP cells *in vitro*. Filipin staining demonstrated that inflammatory cytokine stimulation resulted in free cholesterol accumulation compared with that in unstimulated NP cells (Figure 1G), which was coincident with increases in total cholesterol (to 1.6-fold after TNF-α stimulation and 1.82-fold after IL-1β stimulation) and free cholesterol (to 1.41-fold after TNF-α stimulation and 1.74-fold after IL-1β stimulation), as measured by spectrophotometry (Figure 1H). These results indicated that cholesterol levels were elevated during the progression of IDD, suggesting that cholesterol correlated with IDD.

### Cholesterol Regulated ECM Metabolism in Rat NP Cells

**FIGURE 2:**
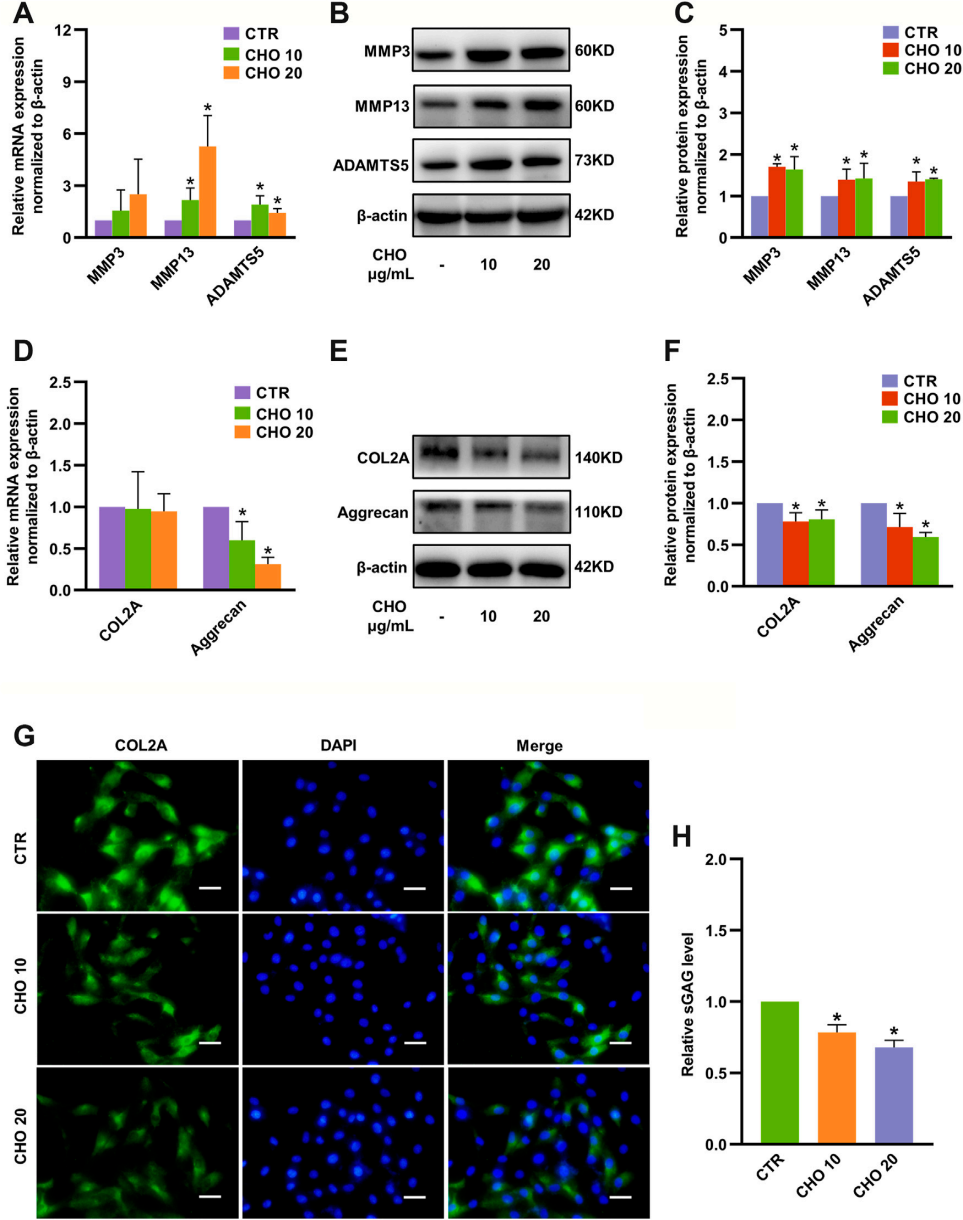
Cholesterol accelerates matrix catabolism and diminishes matrix anabolism in rat NP cells. **(A)** RT-qPCR analysis of MMP3, MMP13 and ADAMTS5 in NP cells treated with 10 and 20 μg/ml cholesterol (n = 3–5). **(B, C)** Western blot analysis and quantification showing that protein expressions of MMP3, MMP13 and ADAMTS5 were markedly upregulated by cholesterol stimulation (n = 3). **(D)** mRNA levels of COL2A and aggrecan in NP cells treated with cholesterol (n = 3–4). **(E, F)** Western blots showing the protein levels of COL2A and aggrecan in NP cells stimulated with 10 and 20 μg/ml cholesterol and the subsequent quantification (n = 3). **(G)** Representative images showing immunofluorescence staining of COL2A in NP cells after different cholesterol treatments (bar = 50 μm). **(H)** The concentrations of sGAG were decreased in the culture media after cells were stimulated with different concentrations of cholesterol for 72 h (n = 4). The data are presented as the mean ± SD. *p < 0.05. CHO, cholesterol.

To evaluate the impact of cholesterol on NP cells, different concentrations of exogenous cholesterol (10 and 20 μg/ml) were administered in NP cells. Gene expression of the ECM degradation-associated proteinases matrix metalloproteinase 13 (MMP13) and ADTAMTS5 was significantly upregulated to 2.15-fold and 1.9-fold by 10 μg/ml cholesterol stimulation and 5.26-fold and 1.46-fold by 20 μg/ml cholesterol stimulation, respectively (Figure 2A). Accordingly, Western blot analysis confirmed that MMP3, MMP13 and ADAMTS5 were markedly increased by cholesterol treatment (Figures 2B,C). Likewise, the mRNA level of aggrecan, a major ECM component, was decreased to 40% by 10 μg/ml cholesterol stimulation and to 70% by 20 μg/ml cholesterol stimulation (Figure 2D). Similarly, the protein levels of COL2A and aggrecan were considerably downregulated to 78 and 71% by 10 μg/ml cholesterol treatment and to 80 and 60% by 20 μg/ml cholesterol treatment (Figures 2E,F). Immunofluorescence analysis also verified that COL2A expression was significantly decreased with the stimulation of cholesterol (Figure 2G). Furthermore, the supernatant levels of glycosaminoglycans (GAGs), another crucial component of the ECM, were prominently diminished by approximately 20 and 30% by 10 and 20 μg/ml cholesterol, respectively (Figure 2H). These results demonstrated that exogenous cholesterol promoted ECM degradation in NP cells.

### Cholesterol Promoted Pyroptosis in Rat NP Cells

**FIGURE 3:**
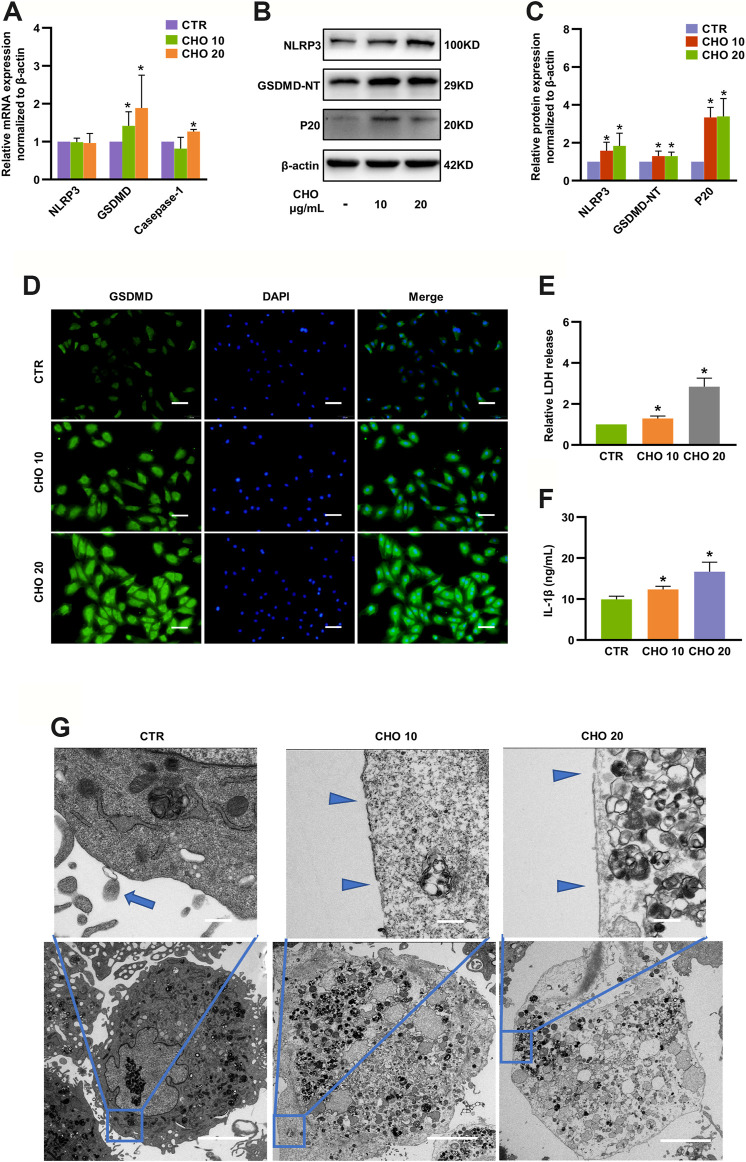
Cholesterol triggers pyroptosis in rat NP cells. **(A)** Analysis of the mRNA levels of NLRP3, GSDMD and Caspase-1 by RT-qPCR (n = 3–4). **(B, C)** Protein expressions of NLRP3, GSDMD-NT and p20 were upregulated upon stimulation with cholesterol for 24 h, as detected by immunoblotting, and were normalized to those of control cells (n = 3–4). **(D)** Immunofluorescence images of NP cells stimulated with cholesterol and stained with an antibody against GSDMD (bar = 100 μm). **(E, F)** LDH and IL-1β release in the culture supernatant was significantly increased in response to increasing concentrations of cholesterol (n = 3). **(G)** TEM images of NP cells (upper panel, bar = 500 nm; lower panel, bar = 5 μm). Control cells showed intact cell membranes with cytoplasmic protrusions (blue arrows). Cholesterol-stimulated cells were characterized by cell swelling and smooth cell membranes with pores (blue triangles). The data are presented as the mean ± SD. *p < 0.05.

Canonical pyroptosis is characterized by activation of NLRP3 inflammasome, GSDMD cleavage and release of IL-1β (Hong et al., 2020). The levels of NLRP3, GSDMD-NT and p20 were markedly increased to 1.58-fold, 1.30-fold and 3.34-fold by 10 μg/ml cholesterol stimulation and 1.84-fold, 1.29-fold and 3.39-fold by 20 μg/ml cholesterol stimulation, although the changes in mRNA expression were only partially consistent with protein expressions (Figures 3A–C). In addition, immunofluorescence analysis also demonstrated that cholesterol treatment significantly increased GSDMD expression in rat NP cells (Figure 3D). To examine the permeability and integrity of the cell membrane, lactate dehydrogenase (LDH) and IL-1β assays were performed. The concentration of LDH in the culture supernatants was significantly elevated by 30% with 10 μg/ml cholesterol treatment and by 180% with 20 μg/ml cholesterol treatment (Figure 3E), and the levels of IL-1β in the culture supernatants were also significantly increased by cholesterol stimulation (9.94 ng/ml in the control group, 12.36 ng/ml in the 10 μg/ml cholesterol treatment group, 16.68 ng/ml in the 20 μg/ml cholesterol treatment group) (Figure 3F). Furthermore, the morphological characteristics of pyroptosis of NP cells were evaluated by TEM, and the results illustrated that control NP cells exhibited normal cell characteristics with well-organized organelles, intact nuclear envelopes and cell membranes with rich cytoplasmic protrusions, while NP cells that were administered with cholesterol exhibited cell swelling, loss of organelles, disappearance of the nucleus and ruptured cell membranes with pores. In summary, cholesterol triggered pyroptotic death in NP cells (Figure 3G).

### An HCD Accelerated IDD in Rats, Which Was Rescued by Atorvastatin

**FIGURE 4:**
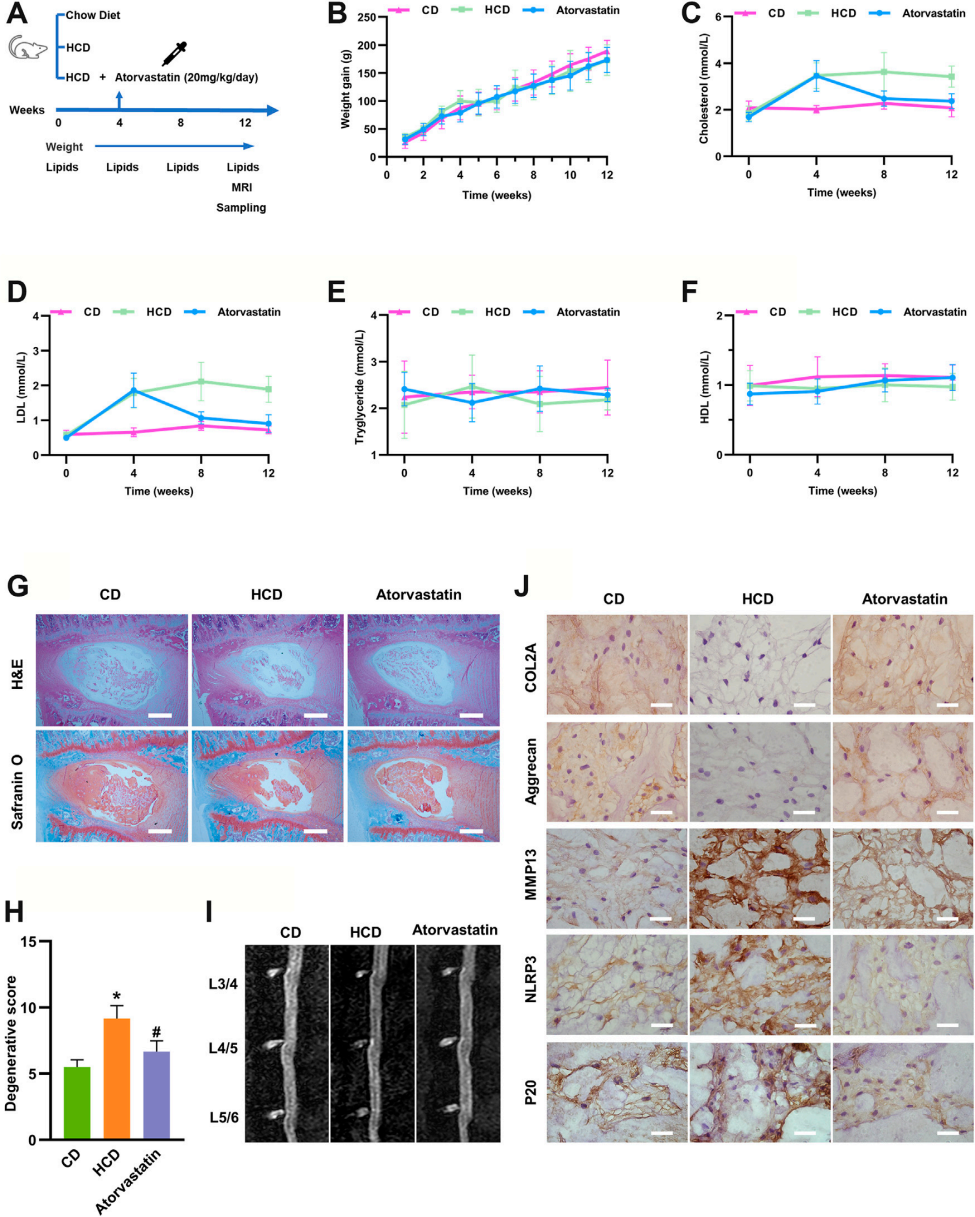
Hypercholesterolemia induces IDD and pyroptosis-associated molecular changes, which were attenuated by atorvastatin. **(A)** Workflow diagram of animal experiments **(B)** Weight gain was not significantly different among SD rats fed different diets at any week (n = 12). **(C, D)** Total serum cholesterol and LDL concentrations of SD rats fed different diets at different weeks (n = 12). **(E, F)** Serum HDL **(E)** and triglyceride concentrations **(F)** of SD rats fed different diets at different weeks (n = 12). **(G**, **H)** H&E and Safranin O/Fast green staining of the lumbar intervertebral discs of SD rats showing that cholesterol induced IDD in the HCD group after 3 months of specific diets and that atorvastatin reversed this effect (bar = 500 μm). *p < 0.05 compared with CD group; ^#^ *p* < 0.05 compared with HCD group. **(I)** Representative sagittal lumbar MRI scans of SD rats fed different diets **(J)** The protein expression levels of the ECM components COL2A and aggrecan, catabolic metabolism marker MMP13, and pyroptosis-associated biomarkers NLRP3 and p20 were measured by IHC (bar = 20 μm). The data are presented as the mean ± SD. CD, chow diet.

Since cholesterol could regulate matrix metabolism and induce cell death *in vitro*, a hypercholesterolemia rat model was established by HCD feeding (containing 2.5% cholesterol) to investigate the role of cholesterol *in vivo*. The cholesterol-lowering drug atorvastatin was also administered 4 weeks after HCD feeding in another group to further confirm whether these effects were cholesterol-related (Figure 4A). Weight gain did not show any significant difference among the three groups at any week (Figure 4B). Similarly, baseline serum total cholesterol and LDL levels were similar in the three groups, whereas rats that were fed an HCD had considerably higher total cholesterol (3.42 ± 0.45 mmol/L) and LDL (1.89 ± 0.37 mmol/L) levels than rats in the control group (total cholesterol: 2.08 ± 0.38 mmol/L, LDL: 0.73 ± 0.11 mmol/L), and these effects were prominently reduced by atorvastatin administration (total cholesterol: 2.38 ± 0.33 mmol/L, LDL: 0.91 ± 0.26 mmol/L) at the end of the experiments (Figures 4C,D). The concentrations of HDL and triglycerides showed no differences among the three groups at any week (Figures 4E,F), indicating that the hypercholesterolemia model was successfully established and atorvastatin reduced cholesterol levels.

Next, we examined whether hypercholesterolemia led to IDD. H&E and safranin-O/fast green staining revealed reduced levels of gelatinous NP tissue and decreases in NP cells in HCD group, while atorvastatin significantly attenuated these effects (Figures 4G,H) after 12 weeks of feeding, though sagittal lumbar MRI images only showed slightly decreased intensity in the intervertebral disc (Figure 4I). Moreover, the immunohistochemistry results suggested that the ECM components COL2A and aggrecan were markedly diminished, whereas the expressions of MMP13 were higher in the HCD group than in the control and atorvastatin groups. Moreover, pyroptosis biomarkers, including NLRP3 and p20, were markedly upregulated in the HCD group and downregulated in the atorvastatin group (Figure 4J). Taken together, these results supported the hypothesis that an HCD significantly contributes to the development of IDD by regulating metabolism of ECM and activating NLRP3 inflammasome *in vivo*.

### Cholesterol Induced Pyroptosis by Modulating ER Stress

**FIGURE 5:**
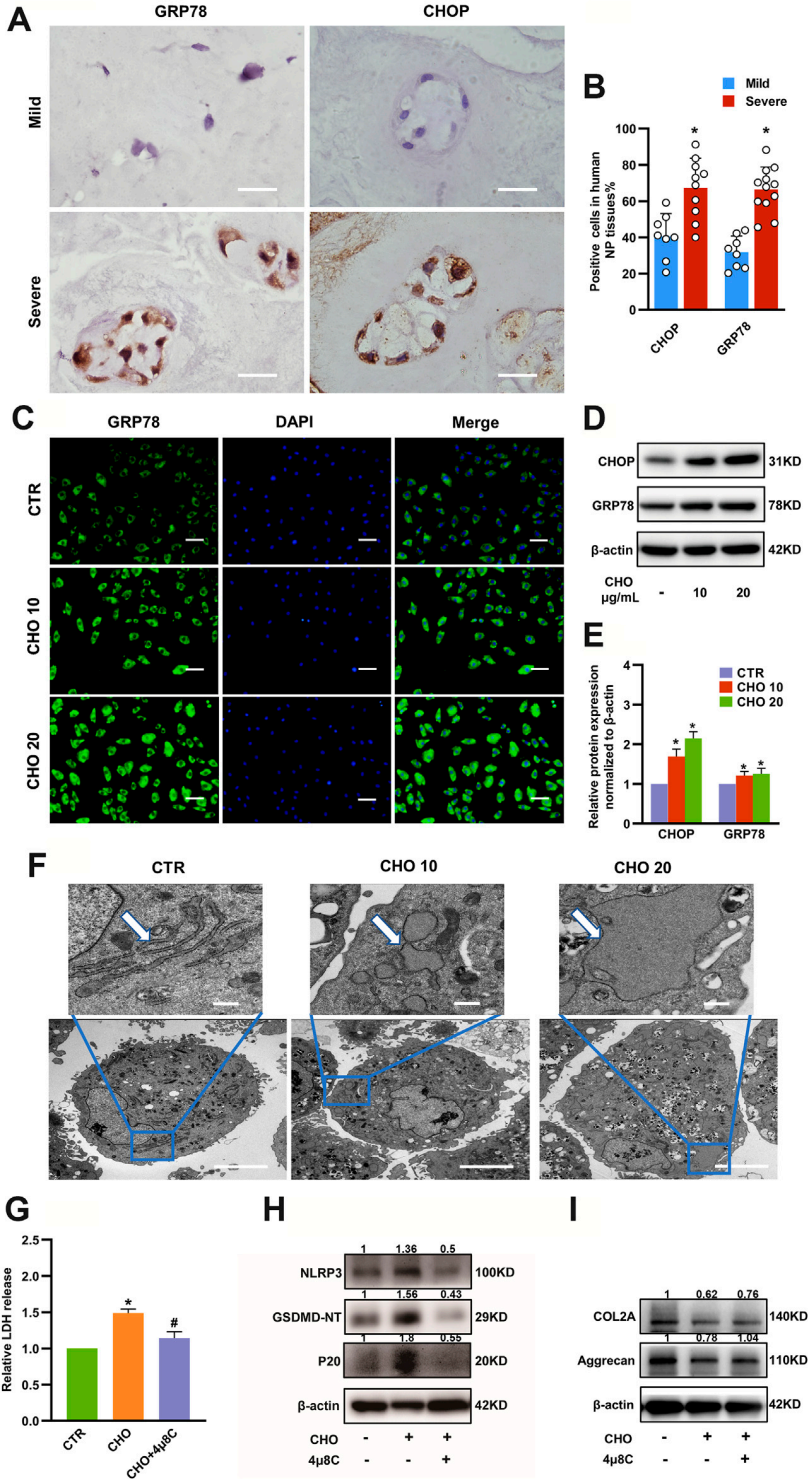
ER stress plays a critical role in cholesterol-induced pyroptosis and matrix degradation in NP cells. **(A)** Representative images of GRP78 and CHOP staining in mild and severe degenerative NP tissues, as determined by IHC (bar = 20 μm). **(B)** Quantification of positive cells showing increased expressions of GRP78 and CHOP in severely degenerative NP tissues (n = 8–12). **(C)** Immunofluorescence images of NP cells stimulated with cholesterol and stained with an antibody against GRP78 (bar = 100 μm). **(D, E)** Protein expression levels of GRP78 and CHOP were upregulated, as revealed by immunoblotting and subsequent quantification (n = 3). **(F)** TEM images of NP cells. Control cells showed slender tube-like rough ERs. Cholesterol-stimulated cells featured swelling and enlarged rough ERs (upper panel, bar = 500 nm; lower panel, bar = 5 μm). **(G)** 4μ8C (20 μM) suppressed cholesterol (10 μg/ml) induced LDH increase in the culture supernatants of NP cells (n = 3). **(H)** Western blot and quantitative analysis showing that the increased pyroptosis biomarkers, including NLRP3, p20 and GSDMD-NT, induced by cholesterol were downregulated by 4μ8C (20 μM) in NP cells. **(I)** Western blot analysis revealed that 4μ8C (20 μM) increased the expression of COL2A and aggrecan in NP cells administrated with 10 μg/ml cholesterol. The data are presented as the mean ± SD. *p < 0.05 compared with control group; ^#^ *p* < 0.05 compared with cholesterol group.

Numerous studies have revealed that ER stress can drive activation of NLRP3 inflammasome and lead to cell death (Lerner et al., 2012; Bronner et al., 2015). To further elucidate whether ER stress was responsible for cholesterol-triggered pyroptosis and affected ECM metabolism in rat NP cells, we first measured the expressions of the ER stress-associated markers GRP78 and CHOP in mild degenerative NP tissues (Grade II and Grade III) and severe degenerative NP tissues (Grade IV and Grade V). IHC results illustrated that the expressions of GRP78 (31.8 ± 8.9% in the mild group vs 66.5 ± 12.4% in the severe group) and CHOP (40.7 ± 12.5% in the mild group vs. 67.3 ± 16.5% in the severe group) were significantly increased in severely degenerative human NP tissues (Figures 5A,B). Immunofluorescence analysis indicated that GRP78 protein expressions were significantly increased in NP cells treated with cholesterol (Figure 5C). Likewise, the protein levels of GRP78 and CHOP were upregulated by 69 and 21% in response to 10 μg/ml cholesterol treatment and by 115 and 25% in response to 20 μg/ml cholesterol treatment (Figures 5D,E). Moreover, the morphological changes of ER were observed by TEM and the results showed increased swelling and dilation of the rough ER in cholesterol-treated NP cells, as indicated by the white arrows (Figure 5F). Subsequently, to determine whether ER stress was responsible for cholesterol-triggered pyroptosis and ECM degradation in NP cells, we used 4μ8C, a small-molecule inhibitor of ER stress, which led to a 20% reduction in LDH release in the presence of cholesterol (Figure 5G). Additionally, 4μ8C obviously attenuated the cholesterol-induced upregulation of NLRP3 (to 37%), GSDMD-NT (to 28%) and p20 (to 30%) (Figure 5H), while COL2A and aggrecan expressions were increased to 1.22-fold and 1.33-fold, respectively, in the 4μ8C plus cholesterol group compared with the cholesterol group (Figure 5I). Collectively, these findings indicated that cholesterol was responsible for ECM degradation and pyroptosis of NP cell by activating ER stress.

### Maturation of SREBP1 Mediated Cholesterol-Induced Pyroptosis and ER Stress in NP Cells

**FIGURE 6:**
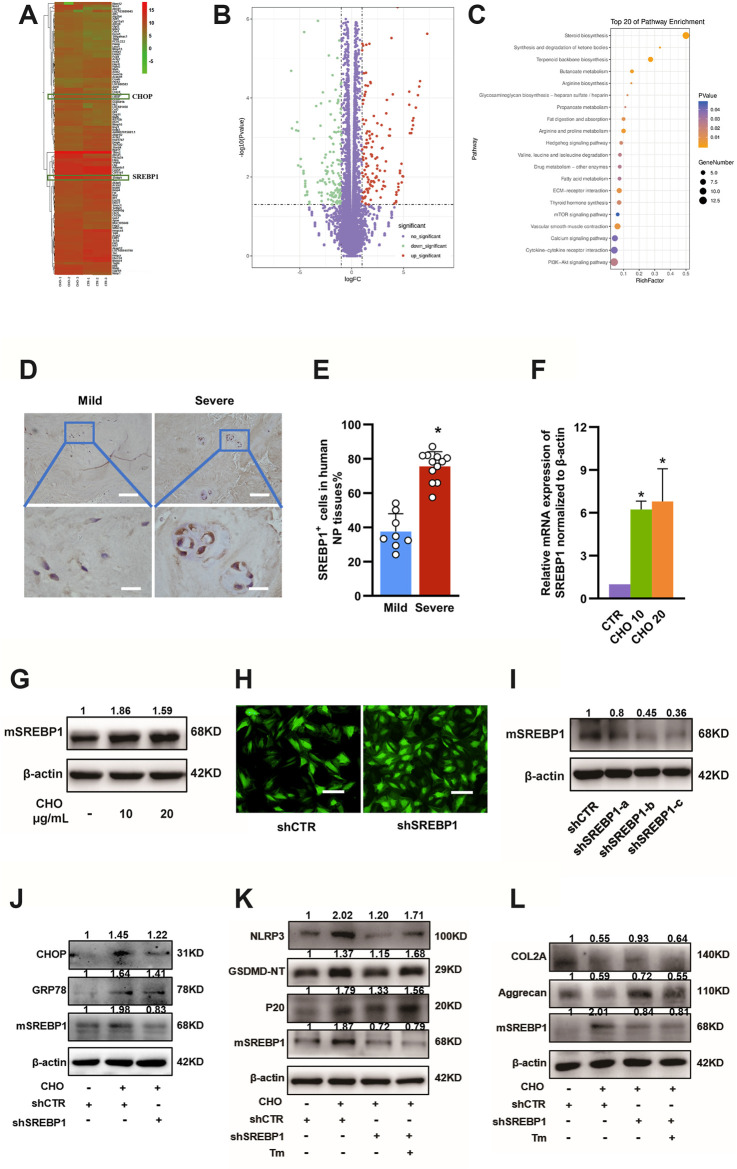
SREBP1 maturation is required for cholesterol-induced ER stress in IDD. **(A, B)** Microarray analysis (heatmaps and volcano plot) of gene expressions of rat NP cells treated with PBS and 10 μg/ml cholesterol is shown. **(C)** Pathway changes in NP cells. **(D)** Representative images of SREBP1 staining in mild and severe degenerative NP tissues, as determined by IHC (upper panel, bar = 100 μm; lower panel, bar = 20 μm). **(E)** Quantification of positive cells showing increased expression of SREBP1 in severe degenerative NP tissues (n = 8–12) **(F)** RT-qPCR analysis of the mRNA levels of SREBP1 showed a significant increase in NP cells treated with cholesterol (n = 3). **(G)** Protein expression levels of mSREBP1 were upregulated by cholesterol stimulation, as determined by immunoblotting **(H)** Immunofluorescence images of NP cells transfected with LV-shCTR and LV-SREBP1 (bar = 100 μm). **(I)** Rat NP cells were transfected with lentiviral vectors containing shRNA-SREBP1 and the shRNA-negative control, and Western blot analysis verified that shRNA-SREBP1 effectively suppressed SREBP1 maturation. **(J)** Western blot showing the effect of SREBP1 knockdown on expression levels of GRP78 and CHOP with stimulation of 10 μg/ml cholesterol in rat NP cells. **(K)** Western blot showing that SREBP1 knockdown decreased pyroptosis-related proteins (NLRP3, GSDMD-NT and p20) in the presence of cholesterol and that Tm (ER stressor, 0.2 μg/ml) reversed this effect. Cells were transfected with the shRNA-negative control, the shRNA-negative control plus 10 μg/ml cholesterol, shRNA-SREBP1 plus 10 μg/ml cholesterol, or shRNA-SREBP1 plus 10 μg/ml cholesterol and Tm. **(L)** Western blot analysis of COL2A and aggrecan revealed the effect of SREBP1 knockdown on anabolic protein expression induced by cholesterol. Cells were transfected with the shRNA-negative control, the shRNA-negative control plus 10 μg/ml cholesterol, shRNA-SREBP1 plus 10 μg/ml cholesterol, or shRNA-SREBP1 plus 10 μg/ml cholesterol and Tm (0.2 μg/ml). The data are presented as the mean ± SD. *p < 0.05.

To investigate how cholesterol induces pyroptosis and ER stress in NP cells, RNA-seq analysis was performed in rat NP cells stimulated with 10 μg/ml cholesterol or PBS. The ER stress-associated gene CHOP was markedly elevated, which was consistent with the results shown in Figure 5D. In addition, NP cells treated with cholesterol showed the most significant changes in lipid metabolism-associated genes compared with control NP cells (Figures 6A–C). Among these significantly altered lipid metabolism-associated genes, sterol regulatory element-binding protein 1 (SREBP1) and its mature form (mSREBP1), which is an important regulator of lipid homeostasis and a transcription factor that regulates the expression of various target gene sets, have been reported to amplify ER stress (Hu et al., 2020). Therefore, we then investigated whether mSREBP1 mediated cholesterol-induced ER stress. The protein level of SREBP1 was considerably higher in severely degenerative human NP tissues (37.6 ± 10.5% in the mild degenerative group vs. 75.7 ± 8.5% in the severe degenerative group), as revealed by IHC (Figures 6D,E). To verify the RNA-seq results, we measured mRNA expressions of SREBP1 and protein levels of mSREBP1 in rat NP cells stimulated with cholesterol. Consistently, 10 and 20 μg/ml cholesterol substantially upregulated gene expressions of SREBP1 to 6.23-fold and 6.80-fold, respectively, and the protein levels of mSREBP1 were considerably elevated by 86 and 59%, respectively (Figures 6F,G), indicating that exogenous cholesterol promoted the maturation of SREBP1.

Next, functional experiments of mSREBP1 were performed using SREBP1 knockdown with lentiviral vectors containing shRNA-SREBP1. The efficacy of SREBP1 knockdown was first measured (Figures 6H,I), and sequence-c was selected for subsequent experiments, as the protein expression of mSREBP1 was decreased by approximately 65%, indicating its capacity to suppress the maturation of SREBP1. To test the effect of SREBP1 maturation on ER stress, the protein expression levels of GRP78 and CHOP were measured by Western blotting, and the results showed that SREBP1 knockdown decreased the protein expressions of GRP78 and CHOP by 16 and 14%, respectively, after cholesterol stimulation (Figure 6J), suggesting that mSREBP1 is required for cholesterol-induced ER stress. To further elucidate whether mSREBP1 is responsible for pyroptosis and ECM degradation by modulating ER stress, we used tunicamycin (Tm) to induce ER stress through accumulation of unfolded proteins in the ER in the presence of cholesterol and SREBP1 knockdown. Western blotting demonstrated that SREBP1 knockdown considerably suppressed the expressions of NLRP3 (to 59%), GSDMD-NT (to 84%) and p20 (to 74%) and markedly increased COL2A (to 169%) and aggrecan (to 122%) in the presence of cholesterol, while Tm reversed the effect of SREBP1 knockdown by increasing the protein levels of NLRP3 (by 71%), GSDMD-NT (by 46%) and p20 (by 17%) and suppressing COL2A (by 31%) and aggrecan (by 24%) expressions (Figures 6K,L), indicating that cholesterol induced pyroptosis and reduced ECM anabolism by activating ER stress through promoting mSREBP1.

## Discussion

In the current study, we first found that metabolism of cholesterol was disturbed and different forms of cholesterol accumulated as IDD progressed. Exogenous cholesterol promoted IDD by mediating the pyroptotic death of NP cells and impairing metabolism of ECM. In addition, Rats fed a HCD exhibited degenerative features in the lumbar intervertebral disc compared with those fed a standard diet, whereas administration of atorvastatin could abolish this effect by lowering cholesterol level. Moreover, cholesterol induced ER stress by promoting the maturation of SREBP1, leading to pyroptosis and ECM degradation. Thus, cholesterol is strongly involved in the development of IDD.

Cholesterol plays a complex and vital role in humans. Dysregulated cholesterol metabolism is attributed to the development and progression of various pathological processes, such as carcinogenesis (Buchwald, 1992), arthrosclerosis (Ference et al., 2017) and osteoarthritis (Sun et al., 2017). In intervertebral discs, NP cells obtain nutrients and expel metabolic waste in harsh environments in which all nutrients and waste are transported by diffusion through cartilaginous endplates (Huang et al., 2014). During aging and disc degeneration, the environment of NP cells becomes even harsher, and extensive alterations occur in nutrient supply, metabolic waste efflux and energy metabolism. Nevertheless, little attention has been given to lipid metabolism, such as that of cholesterol, which is another cardinal nutrient and component of NP cells. Our results demonstrated that cholesterol metabolism was disturbed and different forms of cholesterol were elevated in degenerative NP tissues in both humans and SD rats. Moreover, cholesterol levels increased after NP cells were treated with proinflammatory cytokines, indicating that cholesterol contributed to the severity of IDD. Cholesterol homeostasis is dependent on the balance between uptake, synthesis, storage and efflux, which are precisely controlled by a series of regulators (Luo et al., 2020). Chun et al. reported that total cholesterol, free cholesterol and cholesterol ester levels increased via Lox1-mediated increases in cholesterol uptake in OA chondrocytes (Choi et al., 2019). Therefore, different regulators may be involved in controlling cholesterol metabolism in human and rat NP tissues, leading to different changes in different forms of cholesterol between human and rat NP tissues.

Alterations in cholesterol levels lead to the impairment of cell physiology, such as energy metabolism, biomechanics (Forrester and Griendling, 2019), proliferation (Ghanbari et al., 2020), and signal transduction. Our results demonstrated that exogenous cholesterol could accelerate catabolic metabolism by upregulating MMPs and diminish ECM synthesis by downregulating COL2A, aggrecan and sGAG expression, suggesting dysregulated ECM metabolism in NP cells with stimulation of cholesterol. Similar to the results in chondrocytes, our data revealed that elevated serum cholesterol levels were associated with IDD development independent of body mass index (BMI) as SD rats fed an HCD exhibited degenerative features in the lumbar intervertebral disc compared with those fed a standard diet in a hypercholesterolemia animal model. The effect of cholesterol exposure was further verified by the administration of the cholesterol-lowering drug atorvastatin, which abolished cholesterol-induced ECM degradation. Moreover, atorvastatin diminished OA severity, although both atorvastatin and ezetimibe could reduce serum cholesterol to the same level (Gierman et al., 2014). Our previous and present results also revealed that atorvastatin inhibited the effects of cholesterol and inflammatory cytokines (Chen et al., 2021).

Recent studies have shown that, in addition to apoptosis, pyroptosis is a crucial type of cell death associated with disc degeneration (Li et al., 2021). One study demonstrated that biomarkers of pyroptotic cell death were elevated in degenerative NP tissues compared with normal NP tissues (Xu et al., 2020). Our previous research provided further support for the important role of NLRP3 inflammasome-induced pyroptosis in ECM degradation by showing that NLRP3 knockdown reduced matrix-degrading enzymes, including MMP3 and MMP13, in the presence of TNF-α (Chen et al., 2021). In the present study, exogenous cholesterol was first shown to upregulate expressions of NLRP3, GSDMD-NT and p20, elevate LDH and IL-1β release in the culture supernatant, and induce pore formation on the plasma membrane in NP cells, suggesting that exogenous cholesterol triggered pyroptosis in rat NP cells. Moreover, we confirmed these results in an *in vivo* study and showed that NLRP3 and p20 were upregulated in NP tissues in SD rats fed an HCD, while atorvastatin ameliorated pyroptotic cell death in HCD-fed SD rats. Consistently, our previous study showed that atorvastatin attenuated TNF-α-induced pyroptosis via the NF-κB signaling pathway (Chen et al., 2021). In addition, Guo et al. found that NLRP3 inflammasome activation coupled with the maturation of SREBP2, which is an important cholesterol-regulating molecule (Guo et al., 2018). Additionally, Duewell et al. reported that cholesterol crystals, which are a hallmark of atherosclerotic lesions, were responsible for atherogenesis by activating the NLRP3 inflammasome (Duewell et al., 2010). These studies suggested that both cholesterol itself and its regulators are closely linked with the NLRP3 inflammasome and pyroptosis.

The ER is an important organelle that is responsible for folding proteins, storing calcium and synthesizing lipids (Binet and Sapieha, 2015). Cheng et al. found that hypoxia induced excessive ER stress, leading to NLRP3 inflammasome activation and subsequent GSDMD cleavage, which in turn triggered pyroptotic cell death in the placenta (Cheng et al., 2019). In addition, one study reported that cholesterol disrupted the metabolism of lipids and increased ER stress, inducing CD8^+^ T cell exhaustion in the tumor microenvironment (Ma et al., 2019). Increasing evidence has shown that ER stress is deeply involved in IDD (Liao et al., 2019; Luo et al., 2019). Our study also confirmed the relationship between ER stress and IDD by showing that the ER-related proteins CHOP and GRP78 were elevated in degenerative NP tissues. Moreover, CHOP and GRP78 were also increased in cholesterol-stimulated NP cells, and TEM showed an enlarged ER in NP cells that were treated with cholesterol, indicating that cholesterol could induce ER stress in IDD. In addition, 4μ8C, an ER stress inhibitor, attenuated pyroptosis and promoted ECM production, suggesting that cholesterol-induced pyroptosis in NP cells is ER-dependent.

SREBP1, a lipogenic gene that binds to the rough ER, requires cleavage to release its amino-terminal domain (mSREBP1) in Golgi apparatus, activate its target genes in nucleus and regulate the metabolism of triglycerides (Hagen et al., 2010). Previous studies have reported that SREBP1 can be transformed into mature SREBP1 (mSREBP1) under ER stress (Kim et al., 2019). Moreover, mSREBP1 amplified ER stress by binding to the promoter of PERK (Bronner et al., 2015), indicating a strong relationship between ER stress and SREBP1 maturation. RNA-seq analysis demonstrated that cholesterol disrupted lipid metabolism in NP cells, SREBP1 expression was elevated, and the ER stress-related gene CHOP was upregulated. In addition, SREBP1 knockdown decreased SREBP1 cleavage and maturation and attenuated cholesterol-induced ER stress, pyroptosis and ECM degradation, suggesting that mSREBP1 is required for ER stress and pyroptosis in response to cholesterol stimulation in rat NP cells.

**FIGURE 7:**
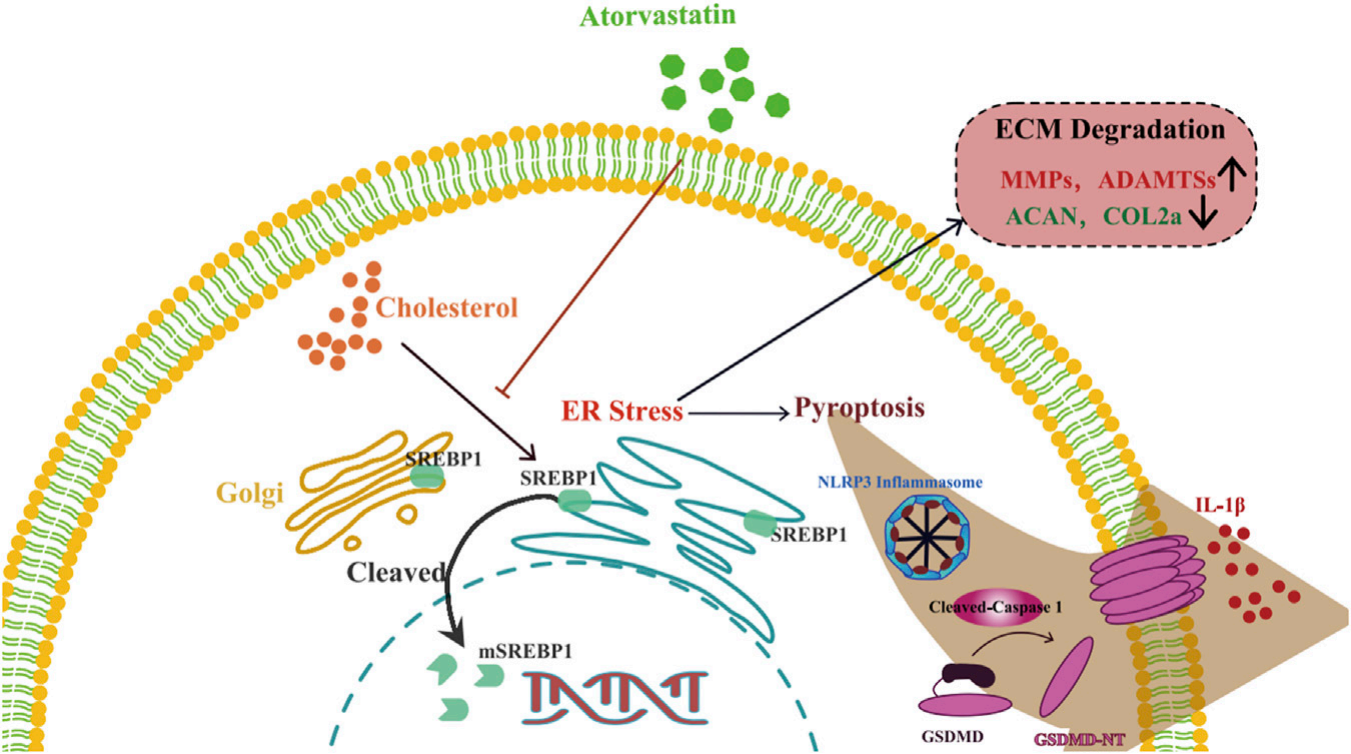
Schematic illustration of the role of cholesterol in the development of IDD. Cholesterol induces pyroptosis of NP cells and ECM degradation by activating endoplasmic reticulum stress through promoting maturation of SREBP1 in the development of IDD.

In summary, our findings showed that cholesterol levels were elevated in degenerative NP tissues. Cholesterol accumulation triggered pyroptosis and promoted ECM degradation in NP cells via mSREBP1-mediated ER stress *in vitro*. In addition, SD rats fed HCDs exhibited more severe IDD than those fed normal diets. This effect could be reversed by the cholesterol-lowering drug atorvastatin. These findings offer a new metabolic pathological mechanism in IDD and a potential therapeutic strategy for the application of atorvastatin to attenuate IDD (Figure 7).

## Data Availability

The data presented in the study are deposited in the Sequence Read Archive (SRA) repository, accession number PRJNA783219.
