# Supplementary material for: Cholesterol Induces Pyroptosis and Matrix Degradation via mSREBP1-Driven Endoplasmic Reticulum Stress in Intervertebral Disc Degeneration
Source: Front Cell Dev Biol. 2022 Jan 31;9:803132. doi: 10.3389/fcell.2021.803132 (PMC8841752; doi:10.3389/fcell.2021.803132)
Supplement: Supplementary file 1 [file Table1.DOCX]

**Supplementary Table 1. Primer sequences used for real‐time PCR**

| **Gene** | **Sense (5’ to 3’)** | **Anti-sense (5’ to 3’)** |
| --- | --- | --- |
| MMP3 | CTAGCAGGTTATCCTAAAAGCATTC | TTGTTTCTTCTCATCAAATCTCCAG |
| MMP13 | TTGTGTGACAGGAGCTAAGGCAGA | GAAGGGGCTAATGAACATGGAGG |
| ADAMTS5 | GAATGTAGACCCTACAGCAACTC | CACACTCCACACTTGTCATACT |
| COL2A | AGCAAGAGCAAGGAGAAGAAGCA | GGACAGTAGACGGAGGAAAGTCA |
| Aggrecan | ACACGGCTCCACTTGATTCTT | CTTGGTCTTTGTGACTCTGCG |
| NLRP3 | GTCCTGAGCCATGGAAGCAA | CTCTGCATGCCGTATCTGGT |
| GSDMD | GGAGGATTTTACAGGACCAGC | GGAGGATTTTACAGGACCAGC |
| Caspase-1 | CCGAGTGGTTCCCTCAAGTT | CAAGACGTGTACGAGTGGGT |
| SREBP1 | TAGTGTTGGCCTGCTTGG | AGGTCAGCTTGTTTGCGAT |
| β-actin | TCTCTGCTCCTCCCTGTTC | ACACCGACCTTCACCATCT |
