# Supplementary material for: Cholesterol Induces Pyroptosis and Matrix Degradation via mSREBP1-Driven Endoplasmic Reticulum Stress in Intervertebral Disc Degeneration
Source: Front Cell Dev Biol. 2022 Jan 31;9:803132. doi: 10.3389/fcell.2021.803132 (PMC8841752; doi:10.3389/fcell.2021.803132)
Supplement: Supplementary file 2 [file Table2.DOCX]

**Supplementary Table 2. Characteristics of patients.**

| **Sample Number** | **Patient Number** | **Degradation**  **Grade** | **Gender** | **Age（year）** | **Usage** |
| --- | --- | --- | --- | --- | --- |
| 1 | 1 | II | Male | 23 | IHC |
| 2 | 2 | II | Female | 30 | IHC |
| 3 | 3 | II | Female | 42 | IHC |
| 4 | 4 | III | Female | 58 | CHO assay, IHC |
| 5 | 5 | III | Male | 33 | CHO assay, IHC |
| 6 | 6 | III | Male | 66 | CHO assay, IHC |
| 7 | 6 | III | Male | 66 | CHO assay, IHC |
| 8 | 7 | III | Female | 57 | CHO assay, IHC |
| 9 | 8 | III | Female | 61 | CHO assay, IHC |
| 10 | 9 | III | Female | 61 | CHO assay, IHC |
| 11 | 10 | III | Male | 68 | CHO assay, IHC |
| 12 | 11 | III | Male | 40 | CHO assay, IHC |
| 13 | 12 | III | Female | 75 | CHO assay, Filipin staining |
| 14 | 13 | III | Male | 64 | Filipin staining, IHC |
| 15 | 13 | III | Male | 64 | Filipin staining, IHC |
| 16 | 14 | III | Female | 59 | Filipin staining, IHC |
| 17 | 15 | IV | Female | 68 | CHO assay, Filipin staining |
| 18 | 16 | IV | Female | 68 | CHO assay, Filipin staining |
| 19 | 17 | IV | Female | 62 | CHO assay, Filipin staining |
| 20 | 18 | IV | Male | 62 | CHO assay, Filipin staining |
| 21 | 19 | IV | Male | 75 | CHO assay, IHC |
| 22 | 20 | IV | Female | 54 | CHO assay, IHC |
| 23 | 21 | IV | Female | 57 | CHO assay, IHC |
| 24 | 22 | IV | Female | 61 | CHO assay, IHC |
| 25 | 23 | IV | Female | 62 | CHO assay, IHC |
| 26 | 24 | IV | Female | 58 | CHO assay, IHC |
| 27 | 24 | IV | Female | 58 | CHO assay, IHC |
| 28 | 25 | IV | Male | 69 | CHO assay, IHC |
| 29 | 25 | IV | Male | 69 | IHC |
| 30 | 26 | IV | Male | 37 | IHC |
| 31 | 27 | V | Male | 71 | CHO assay, Filipin staining |
| 32 | 27 | V | Male | 71 | CHO assay, Filipin staining |
| 33 | 28 | V | Male | 62 | CHO assay, Filipin staining |
| 34 | 29 | V | Male | 68 | CHO assay, Filipin staining |
| 35 | 30 | V | Female | 59 | CHO assay |
| 36 | 31 | V | Female | 67 | CHO assay |
| 37 | 32 | V | Male | 54 | CHO assay |
| 38 | 33 | V | Female | 72 | CHO assay, IHC |
| 39 | 34 | V | Male | 62 | CHO assay, IHC |
| 40 | 34 | V | Male | 62 | CHO assay, IHC |

CHO, cholesterol; IHC, immunohistochemistry staining.
